# Loss of Myoferlin Redirects Breast Cancer Cell Motility towards Collective Migration

**DOI:** 10.1371/journal.pone.0086110

**Published:** 2014-02-26

**Authors:** Leonithas I. Volakis, Ruth Li, William E. Ackerman, Cosmin Mihai, Meagan Bechel, Taryn L. Summerfield, Christopher S. Ahn, Heather M. Powell, Rachel Zielinski, Thomas J. Rosol, Samir N. Ghadiali, Douglas A. Kniss

**Affiliations:** 1 Department of Biomedical Engineering, The Ohio State University, Columbus, Ohio, United States of America; 2 Department of Obstetrics & Gynecology (Division of Maternal-Fetal Medicine and Laboratory of Perinatal Research), The Ohio State University, Columbus, Ohio, United States of America; 3 Department of Material Science Engineering, The Ohio State University, Columbus, Ohio, United States of America; 4 Department of Veterinary Biosciences, College of Veterinary Medicine, The Ohio State University, Columbus, Ohio, United States of America; 5 The Dorothy M. Davis Heart and Lung Research Institute, The Ohio State University, Columbus, Ohio, United States of America; Sun Yat-sen University Medical School, China

## Abstract

Cell migration plays a central role in the invasion and metastasis of tumors. As cells leave the primary tumor, they undergo an epithelial to mesenchymal transition (EMT) and migrate as single cells. Epithelial tumor cells may also migrate in a highly directional manner as a collective group in some settings. We previously discovered that myoferlin (MYOF) is overexpressed in breast cancer cells and depletion of MYOF results in a mesenchymal to epithelial transition (MET) and reduced invasion through extracellular matrix (ECM). However, the biomechanical mechanisms governing cell motility during MYOF depletion are poorly understood. We first demonstrated that lentivirus-driven shRNA-induced MYOF loss in MDA-MB-231 breast cancer cells (MDA-231^MYOF-KD^) leads to an epithelial morphology compared to the mesenchymal morphology observed in control (MDA- 231^LTVC^) and wild-type cells. Knockdown of MYOF led to significant reductions in cell migration velocity and MDA- 231^MYOF-KD^ cells migrated directionally and collectively, while MDA-231^LTVC^ cells exhibited single cell migration. Decreased migration velocity and collective migration were accompanied by significant changes in cell mechanics. MDA-231^MYOF-KD^ cells exhibited a 2-fold decrease in cell stiffness, a 2-fold increase in cell-substrate adhesion and a 1.5-fold decrease in traction force generation. *In vivo* studies demonstrated that when immunocompromised mice were implanted with MDA- 231^MYOF-KD^ cells, tumors were smaller and demonstrated lower tumor burden. Moreover, MDA- 231^MYOF-KD^ tumors were highly circularized and did not invade locally into the adventia in contrast to MDA- 231^LTVC^-injected animals. Thus MYOF loss is associated with a change in tumor formation in xenografts and leads to smaller, less invasive tumors. These data indicate that MYOF, a previously unrecognized protein in cancer, is involved in MDA-MB-231 cell migration and contributes to biomechanical alterations. Our results indicate that changes in biomechanical properties following loss of this protein may be an effective way to alter the invasive capacity of cancer cells.

## Introduction

Cell migration is an essential biological process involved in inflammation, tissue repair and regeneration, developmental events, cancer, and immune cell surveillance. In many instances, individual cells migrate within the extracellular matrix (ECM) in a polarized manner, extending forward lamellipodia and actin-rich filopodia [1], [2] via either protease-dependent or independent mechanisms [3]. In combination with these cellular protrusions, focal adhesion dynamics, actin polymerization, and actomyosin contraction result in internal tension within the cell. This tension can promote stress fiber formation and enhance mechano-signaling [4]. During single cell migration, the formation of distinct leading and trailing edges coordinate migration activity [5], while collective cell migration is governed by several biophysical factors including the distribution of tensile stress within the monolayer [6], transmission of mechanical force across cell-cell junctions [6], [7], and the distribution of cell stiffness within the advancing cell sheet [8]. In both cases, the cell motility cycle involves steps that occur in many cell types in response to external stimuli and to intracellular and intercellular signaling [9]. These steps include establishing cell polarity by intracellular signaling events that direct leading edge protrusions, integrin-mediated adhesions and focal adhesion development, cytoskeleton remodeling, and directed contraction and detachment at the rear of the cell [10]–[12]. In addition, migrating cells can be quite versatile, and can switch between enzyme- and non-enzyme- driven methods of movement depending upon their local microenvironmental terrain [13]. Biochemical and mechanical signals promote complex cellular interactions with the ECM and provide tumor cells with the ability to deform, degrade, and remodel the ECM to proficiently migrate and invade. This interaction between the tumor and stroma cells with the ECM also represents a primary factor in epithelial to mesenchymal transition (EMT) [14].

EMT is a biological program exemplified during embryogenesis, fibrosis and wound repair, and cancer metastasis [15]. In cancer, EMT represents a transdifferentiation program induced by transcription factors, including Snail 1, Snail 2, Twist, Zeb 1 and Zeb 2, in epithelial cells generating mesenchymal traits for metastasis [16]–[18]. Epithelial cells, that are typically sessile, undergo an EMT as they adopt a fibroblastic or amoeboid phenotype and become highly migratory after expressing a complex EMT gene program [14], [16]. During EMT, epithelial cells lose direct cell-cell contact by degrading E-cadherin and other intercellular junction proteins, allowing them to migrate away from their local neighborhood and into surrounding tissue stroma [19].

In contrast to the acquisition of single-cell motility after loss of junctional proteins in mesenchymal cells, epithelial cells maintain cell-cell junctions and move as cohesive, communal cell sheets, clusters or threads [5]. Collective migration occurs during embryogenesis, re-epithelialization during cutaneous wound healing, and in cancer invasion [20]. Although it was originally thought that contractile force in cells at the leading edge of a cell monolayer (leader cells) was the primary mechanisms of collective cell migration, Trepat and colleagues [6] demonstrated that cells located far from the leading edge generate contractile forces and that this leads to tensile stress within the monolayer. Furthermore, Trepat also demonstrated that stress within the monolayer governs the direction of cell movement [7]. Interestingly, these authors noted that after EMT, cells do not conform to this behavior and have a more widely distributed migration pattern.

While the biological and biomechanical characteristics of single-cell and collective migration have been described in some detail, there is still relatively little information about the molecular and biomechanical mechanisms that govern EMT and/or the reverse the mesenchymal-to-epithelial transition (MET). We recently discovered that silencing a protein involved in membrane dynamics, myoferlin (MYOF), in the highly invasive MDA-MB-231 breast cancer cell line induces MET and modulates the invasive capacity of these cells [21]. MYOF is part of the ferlin family of proteins that are involved in membrane dynamics, vesicle trafficking, cell motility, and maintenance of plasma membrane integrity. Originally discovered in *Caenorhabditis elegans* (FER-1), ferlin deficiency was originally correlated with loss of migration in the *C. elegans* sperm cells and reduced vesicle fusion necessary for amoeboid migration [22]. Mammalian ferlins are associated with several vesicle fusion events, including plasma membrane repair and synaptic vesicle fusion to the presynaptic membrane [23]. MYOF is important in skeletal myogenesis [24] and endothelial cell membrane repair and stabilization of several receptor tyrosine kinases [25], [26].

Although our previous study demonstrated that silencing MYOF reduced the invasive capacity of MDA-MB-231 cells, techniques used in that study did not allow us to investigate transitions between collective and single cell motility. Therefore, in the current work, we used short-hairpin loop-based RNA interference (shRNAi) and live-cell imaging and tracking techniques to investigate how silencing MYOF in MDA-MB-231 cells influences transitions between collective and single cell migration. In addition, since there is very little information about the biomechanical mechanisms responsible for transitions between mesenchymal and epithelial motility patterns, we also used a battery of biomechanical characterization tools to investigate how cellular biomechanical properties are modulated during the MET. Finally, our *in vitro* results suggested that silencing MYOF can significantly alter the biomechanics of tumor growth and morphogenesis. To test this *in vivo*, we used a mouse xenograft model to investigate how MYOF loss would affect tumor formation and local invasion.

## Materials and Methods

### Ethics Statement

All animal experiments were conducted in accordance with procedures approved by the Institutional Animal Care and Use Committee (IACUC) at The Ohio State University (Protocol # 2009A0099).

### Cell Culture and Generation of shRNA Lentiviral Constructs

The highly invasive human breast cancer cell line MDA-MB-231 (ATCC, HTB-26) derived from a pleural effusion was used throughout the studies described in this manuscript (the parental line is referred to as MDA-231^WT^). Stable lines of MDA-MB-231 cells expressing short hairpin loop structures encoding interfering RNAs directed against human myoferlin (designated MDA-231^MYOF-KD^) or a non-human, non-targeting control shRNA (designated MDA-231^LTVC^) were generated using lentiviral-based delivery (MISSION™, Sigma-Aldrich, St. Louis, MO) (see Figure S1). All MDA-MB-231 cell lines were maintained in high-glucose (4.5 g/l) Dulbecco’s Modified Eagle Medium (DMEM) supplemented with 10% fetal bovine serum (FBS, Invitrogen, Carlsbad, CA).

### Scanning Electron Microscopy

MDA-231^WT^, MDA-231^LTVC^, and MDA-231^MYOF-KD^ cells (2.5×10^4^/well) were seeded onto (polyethylene terephthalate, PET) coverslips (Thermanox™ disks, NUNC, Rochester, NY) and incubated (37°C, 5% CO_2_, saturated humidity) for 12 h. Cells were fixed with 3% glutaraldehyde (Sigma-Aldrich, St. Louis, MO), post-fixed with 1% OsO_4_ and dehydrated with an ethanol and hexamethyldisilizane (HMDS, Electron Microscope Sciences, Hatfield, PA) dilution series. After mounting on scanning electron microscopy stubs, samples were prepared for scanning electron microscopy using a Pelco Model 3 sputter coater with gold-palladium (0.07 mbar, 17 mA, 110 sec). Samples were imaged in an FEI Nova NanoSEM microscope equipped with secondary electron mode at 5 kV. All scanning EM specimens were stored at room temperature in a desiccator to maintain specimen integrity.

Morphometric analysis was performed using ImageJ to determine cell surface area and the number and length of lamellipodia and filopodia [27]. Quantification of filopodia number was performed by measuring the filopodial length. Quantification of lamellipodia number and morphology was performed by calculating the surface area and the number of lamellipodia for each cell evaluated. Finally, the total cell surface area was also calculated. For these morphometric measurements, at least 30 cells of each type were analyzed and data were expressed as mean ± standard error of the mean (SEM) and statistically evaluated by one-way analysis of variance followed by Tukey *post hoc* testing (p<0.05 considered significant).

### Live-cell Microscopy

Cultures of MDA-231^LTVC^ and MDA-231^MYOF-KD^ cells were rinsed with PBS, trypsinized and resuspended in 10% FBS-containing media at 3×10^5^ cells/ml. Cell suspensions (80 µl) were placed into both chambers of a wound assay insert (Ibidi®, Martinsried, Germany) which allows for the formation of a well-defined “wound edge” without scratching the monolayer. MDA-231^MYOF-KD^ and MDA-231^LTVC^ cells were incubated (37°C, 5% CO_2_/95% air) for 24 h until a nearly confluent monolayer was formed. On the next day, the insert was removed and 1.5 ml of complete medium was added to the culture dish that was placed in a live-cell imaging system (self-contained, humidified, temperature and CO_2_-controlled incubator outfitted on the stage of a Zeiss Axiovert 200M microscope, assembled by Marianas 3i, Denver, CO). Images were captured using a 10X objective every 10 min over a 24-h time period. Experiments were repeated three times and 100 cells were evaluated per run using the ImageJ Manual Tracking feature. Image processing was carried out using the Ibidi Chemotaxis and Migration Plugin for ImageJ 1.01 (Ibidi.com/software/chemotaxis_and_migration_tool). We first used this plugin to compute mean cell velocity according to:
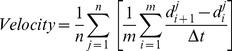
(1)where n is the total number of cells per experimenta (n = 100), m is the number of time intervals for each cell (typically 145 time intervals over 24 hours), d_i_^j^ is the distance traveled for cell “j” at timepoint “i”, and Δt is the time interval between images (typically 10 min). Next, the plugin was used to calcuate the mean cell directionality according to:
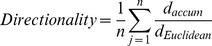
(2)where d_*Euclidean*_ is the distance between the initial and final timepoints for each cell, d_*accum*_ is the accumulated distance between each successive timepoint during the 24-h period for each cell type, and n = total number of cells analyzed per wound. Directionality values near unity indicate a highly directional movement in a straight line typical of collective migration, while values near zero indicate random movement of the cell typical of single cell migration.

### Cell Adhesion and Spreading

Cells were seeded into 16-well E-plates and an *xCELLigence*™ real time cell analyzer platform (RTCA, ACEA Biosciences, Inc.) was used to evaluate relative cell adhesion and spreading of MDA-231^MYOF-KD^, MDA-231^LTVC^, and MDA-231^WT^ cells. E-plates were equilibrated with 100 µl of medium containing 1% or 10% FBS prior to seeding cells. After correcting for background impedance, cells (4×10^4^/well) were seeded in a final volume of 100 µl of medium containing either 1% or 10% FBS. Cells settled onto the surface of the well for 30 min and E-plates were then placed into the RTCA chamber within a humidified incubator (37°C, 5% CO_2_/95% air) for data collection. Relative impedance, expressed as cell index (CI, see equation 3), was measured every 5 min for 4 h. Experiments were repeated 3 times with at least 4 replicates per cell type in a given run.
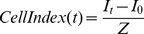
(3)where *I_t_* is the impedance measured at a discrete frequency at a given time point during the experiment (i.e., after cell seeding) and *I_0_* is the impedance measured at the same frequency before cells were added. Z is the frequency factor in Ohms (Ω) at the relevant frequency. xCELLigence measures at 3 discrete frequencies and therefore there are 3 corresponding Z factors: 10 kHz [15 Ω], 25 kHz [12 Ω], and 50 kHz [10 Ω]. Thus, at the same seeding density, without significant cell proliferation during short incubations, cells with higher cell adhesion and/or degree of spreading manifest a higher CI. Cell Index readings were normalized to the average MDA-231^WT^ CI value in each experiment. The relative adhesion and spreading (RAS) for each replicate was calculated as follows:



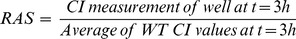
(4)The RAS values for each cell type were combined across all experiments and plotted as the mean ± SEM.

### Adhesion Strength Measurements

E-plates and the xCELLigence RTCA were used for the evaluation of cell adhesion using a novel real-time trypsin release assay developed by our laboratory. E-plates were equilibrated with 100 µl of 1% FBS-containing DMEM. After background impedance was measured, 4×10^4^ cells/well were seeded in a volume of 100 µl of 1% FBS. After a 30-min pre-equilibration, E-plates were placed into the RTCA chamber and cells were grown for 2 days during which CI was recorded at 30-min intervals. To initiate cell release, 10 µl of 0.41% trypsin/EDTA were added to each well of the E-plate (final concentration of 0.02% trypsin/EDTA) and the CI was monitored at 10-sec intervals for 2 h at 37°C. Experiments were repeated 3 times with at least 4 replicates per cell type per run. The CI decreased as cell-substrate contacts were released, and are plotted as percent of the CI reading just prior to the addition of trypsin to correct for cell number and cell spreading effects on CI. Wells with outliers at any time point as determined by non-linear one-phase exponential decay regression analysis (GraphPad Prism) were excluded from further analysis. The remaining data (n ≥10 per cell type) were fitted with a non-linear one-phase exponential decay curve with a constraint of y_0_ = 100%.

### Atomic Force Microscopy

In this study, we used a previously described [8] atomic force microscopy (AFM) technique to evaluate changes in cell stiffness. MDA-231^LTVC^ and MDA-231^MYOF-KD^ cells were seeded at 5–8×10^5^ cells in 60-mm cell culture dishes and grown to ∼80–90% confluence in complete medium. Prior to AFM analysis, the culture medium was replaced with CO_2_-independent medium (Invitrogen, Carlsbad, CA) supplemented with 4 mM L-glutamine, 1% FBS, and 1% penicillin/streptomycin. A Bioscope II AFM (Veeco, Santa Barbara, CA) mounted on the stage of a Zeiss Axiovert 200 inverted optical microscope was used in tapping mode for imaging. All samples were imaged in the supplemented CO_2_-independent medium using a fluid cell model. Silicon nitride (SiN) triangular cantilevers (Veeco) 200 µm in length with a tip angle θ = 35° and a nominal spring constant *k* of 0.01 N/m were used to image and determine the Young’s modulus (a measure of cell stiffness) as described previously [8]. Briefly, the AFM tip was moved in the vertical direction (z) towards the cell surface and the tip deflection (d) was recorded as a function of z. Hooke’s law (F = k•d) was used to generate a force/displacement curve which was analyzed with the following modified Hertz model [28]–[30]:
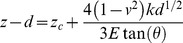
(5)where E and ν are the Young’s modulus and the Poisson’s ratio, respectively, and z_c_ is the contact point. Assuming a ν = 0.5 (i.e., an incompressible material), E and z_c_ were estimated by least-squares fitting of Eq. 5 with the experimentally measured deflection (d) as function of z. Approximately 1500 force curves were analyzed for each cell type and analysis was restricted to indentations less than 800 nm.

### Traction Force Microscopy

We used traction force microscopy (TFM) to evaluate changes in cell contractility. Polyacrylamide substrates with a Young’s modulus of 5.9 kPa, measured by indentation AFM, were prepared on 25 mm glass coverslips using 40 µl of an 8% acrylamide, 0.04% N,N’-methylene-bis-acrylamide solution (BioRad, Hercules, CA), and embedded with a 0.01% suspension of 0.5 µm diameter red fluorescent carboxylate-modified beads (Invitrogen, Carlsbad, CA). The surface of gels was functionalized using Sulpho SANPAH (Thermo Scientific, Waltham, MA), and coated with 0.2 mg/ml of bovine collagen type I (Advanced BioMatrix Inc, San Diego, CA). Cells were seeded sparsely (∼1000 cells/gel) and allowed to adhere overnight. Phase contrast images of cell boundaries were obtained for adhered cells, and fluorescent images of bead positions were obtained before and after cell detachment with trypsin. Image registration and tracking of bead displacements was computed using correlation-based particle image velocimetry (MATLAB, MathWorks, Natick, MA). Displacements were then applied as a boundary condition on the surface of the gel, and the traction stress field on the gel surface was calculated using the COMSOL finite element package (COMSOL Multiphysics, Burlington, MA) as described previously [31]. This analysis resulted in a spatial map of the traction stresses, T(x,y), exerted by the cell on the substrate. This traction stress field was used to calculate the average traction stress exerted by an individual cell according to:
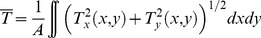
(6)where T_x_ and T_y_ represent tractions in the x and y directions, A is the surface area of cell-substrate interaction and 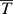 is a measure of the overall contractility of the cell.

### In vivo Murine Xenograft Model of Tumor Formation

In this study we used a xenograft model of tumor formation to investigate how silencing MYOF influences tumor growth *in vivo*. All animal experiments were conducted in accordance with procedures approved by the Institutional Animal Care and Use Committee (IACUC) at The Ohio State University (Protocol # 2009A0099). Female nude mice (Taconic NCRNU-F athymic homozygous) were housed under barrier conditions in ventilated cages with sterile rodent chow and water available *ad libitum*. For xenotransplantation, MDA-231^LTVC^ and MDA-231^MYOF-KD^ cell suspensions (3×10^6^ cells/100 µl of 50∶50 PBS-Matrigel) were injected subcutaneously into nude mice (n = 5 mice per group), tumor growth was monitored with caliper measurements over 7 weeks. At 7 weeks, mice were euthanized and tumors were dissected, weighed, and fixed with 10% neutral-buffered formalin for 24 h. Next, the samples were dehydrated in a graded ethanol series and embedded in paraffin. Sections (5–6 µm) were cut and placed onto clean glass microscope slides. After de-paraffinizing with xylene, sections were stained with hematoxylin and eosin (H&E), dehydrated and mounted.

For immunolabeling, antigen retrieval was accomplished by incubating de-paraffinized sections in 10 mM sodium citrate (pH 6.0) with 0.05% Tween-20 in an autoclave for 4 min at 18 psi and 122°C. Sections were then incubated with rabbit polyclonal anti-human myoferlin (*Prestige* 1∶350, Rabbit Anti-Human Myoferlin HPA014245, Sigma-Aldrich, St. Louis, MO) using the UltraVision LP Detection System (Thermo Scientific, Fremont, CA), according to the manufacturer’s specifications. Following histological staining, slides were digitized using the Aperio ScanScope system (Aperio, Vista, CA) by the Comparative Pathology and Mouse Phenotyping Shared Resource of The Ohio State University College of Veterinary Medicine.

Additional measurements included tumor burden (by percent total body weight), final tumor mass, and tumor circularity calculated as:
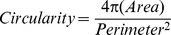
(7)


### Statistical Analysis

Statistical analyses were performed using Prism 5.0 (GraphPad). The distributions of all variables were first tested using D-Agostino & Pearson omnibus normality testing (GraphPad Prism) to determine whether parametric or non-parametric statistical testing was appropriate in each case. Statistically significant differences were then determined using either the two-tailed Student’s t-test, the Mann-Whitney U test, Krustal Wallis test followed by Dunn’s *post-hoc* test, one-way analysis of variance (ANOVA) followed by Tukey’s *post-hoc* test, or two-way ANOVA with Bonferonni *post hoc* testing, where appropriate (as indicated in the text and figure legends). Statistical significance was considered at a minimum value of p<0.05.

## Results

### Myoferlin-depleted MDA-MB-231 Cells Undergo a Morphological Transition and Show Altered Leading Edge Protrusions

**Figure 1:**
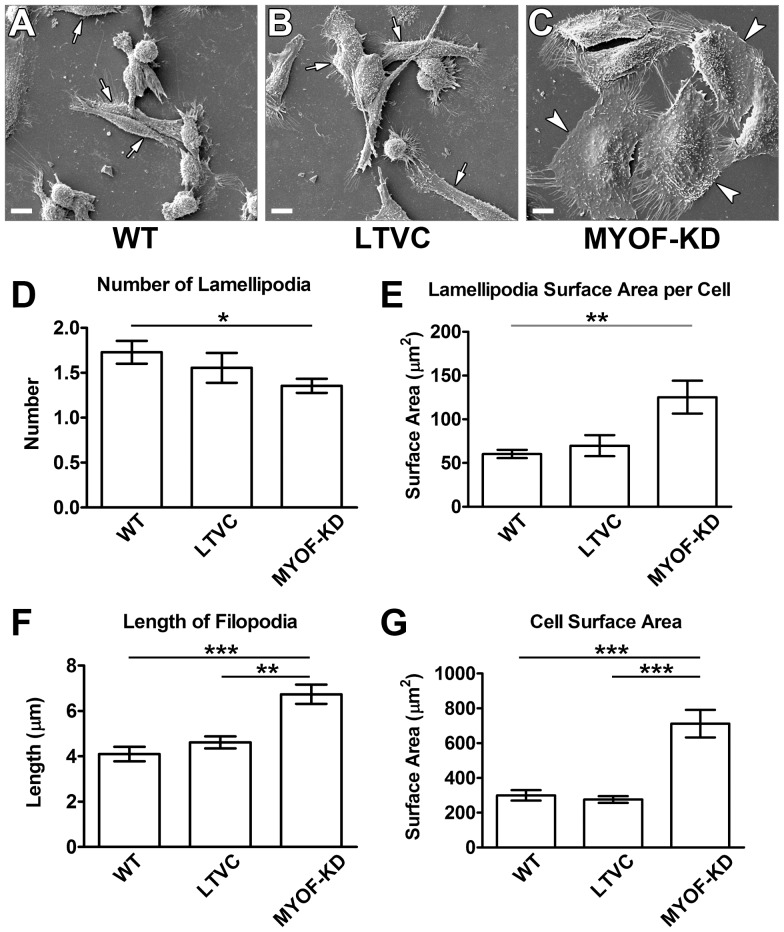
MYOF depletion in MDA-MB-231 cells alters overall morphology and the appearance of leading edge protrusions. (A–C) SEM images of (A) wild-type (MDA-231^WT^), (B) MDA-231^LTVC^, and (C) MDA-231^MYOF-KD^ cells when seeded at subconfluent concentrations (∼30%). Note the mesenchymal-like morphology of the MDA-231^WT^ and MDA-231^LTVC^ cells (arrows) compared to the more epithelial morphology of MDA-231^MYOF-KD^ cells (arrowheads). (D–G) Morphometric analyses of all MDA-MB-231 cell lines. Data included measurements on: (D) lamellipodia number per cell, (E) lamellipodia surface area per cell, (F) filopodia length, and (G) cell surface area. Bars represent mean ± SEM. Statistical significance was between the starred bar and the other two cell types shown (Kruskal Wallis with Dunn’s multiple comparison test), where *p<0.05, **p<0.01, and ***p<0.001. Scale bars = 10 µm.

Using scanning electron microscopy, we evaluated the morphology (Figure 1A, B, and C) of MDA-MB-231 human breast tumor cells before (MDA-231^WT^) and after (MDA-231^MYOF-KD^) stable lentivirus-mediated transduction of shRNAs targeting MYOF as well as in a control line expressing a non-human, non-targeting shRNA (MDA-231^LTVC^). The parental MDA-231^WT^ and MDA-231^LTVC^ cell lines exhibited a mesenchymal phenotype with an average of 1.5–1.8 lamellipodia per cell and a lamellipodia surface area of ∼ 50 µm^2^ per cell (Figure 1A, B, D, and E). In contrast, when MYOF was diminished by >90%, cells reverted to an epithelial shape that was polygonal and typically contained a single broad lamellipodium with longer filopodia (Figure 1C and F). In addition, the average surface area of MYOF-depleted MDA-MB-231 cells was more than two-fold greater than either wild-type or MDA-231^LTVC^ cells (Figure 1G).

### Myoferlin Alters Cell Migration Properties of MDA-MB-231 Cells

**Figure 2:**
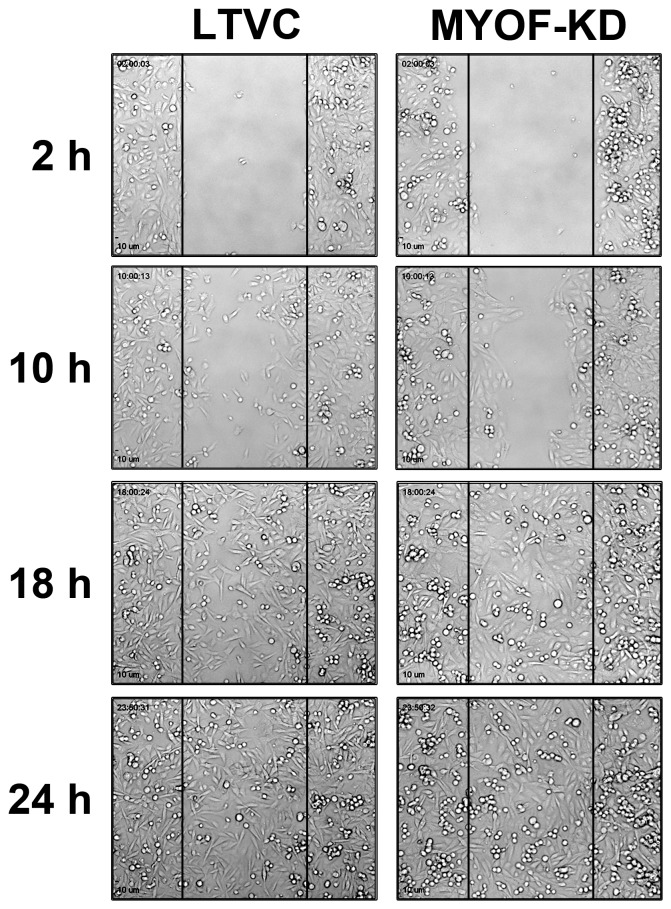
MYOF depletion of MDA-MB-231 cells promotes adoption of a collective mode of migration. Representative phase contrast time-lapse images of MDA-231^LTVC^ and MDA-231^MYOF-KD^ cells taken during 2D wound closure bioassays. Images were collected every 10 min for 24 h following removal of the Ibidi® chamber inserts; four of these time points are demonstrated. Note that while the MDA-231^LTVC^ cells exhibited a random pattern of individual cell migration, the MDA-231^MYOF-KD^ cells moved in a collective pattern reminiscent of epithelial cell migration. Scale bars = 10 µm. See Video S1 (MDA-231^LTVC^) and S2 (MDA-231^MYOF-KD^) for movies of cell migration over 24 h.

**Figure 3:**
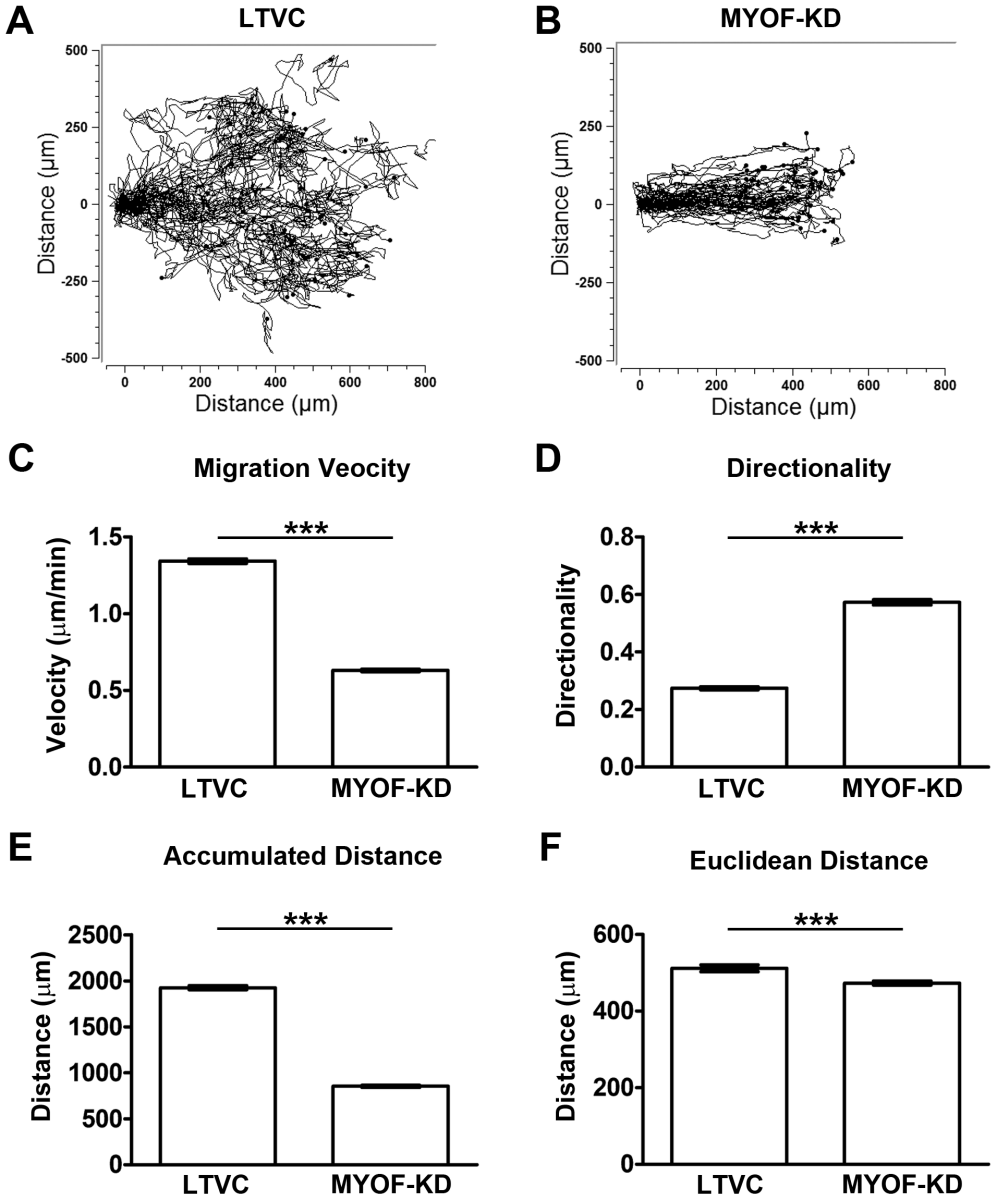
MYOF-depleted MDA-MB-231 cells exhibit reduced average migration velocity but increased directional persistence. Cell track samples of 50 cells from (A) MDA- 231^LTVC^, and (B) MDA- 231^MYOF-KD^. Wound assay migration properties of MDA-MB-231 cells: (C) Velocity, (D) Directionality, (E) Total Distance Traveled (Path Dependent Distance), (F) Path Independent Distance Traveled (as described in Materials and Methods). For each of these properties, 300 cells of either type were tracked over 24 h in 3 independent experiments. Bars represent mean ± SEM. Statistical significance was between the starred bar and the other two cell types shown (Kruskal Wallis with Dunn’s multiple comparison test), where ***p<0.001.

We used an Ibidi wound healing assay and live-cell imaging and tracking techniques to assess the cell migration properties of MDA-231^MYOF-KD^ and MDA-231^LTVC^ cells. Cells were seeded into wound assay chambers and grown to near confluence followed by removal of the insert and real-time imaging. Unlike standard scratch-wound healing assays, this technique does not induce a “scratch” injury to the monolayer and the tracking of live-cells allows for a more detailed and precise analysis of cell migration as compared to standard wound healing assays. For example, although it appeared that MDA-231^LTVC^ control cells and MDA-231^MYOF-KD^ cells closed the gap created by the Ibidi insert within 24 h (Figure 2), further analysis of the data revealed a substantial shift in the mode of motility during this period. This was illustrated by examining the manner by which MDA-231^LTVC^ and MDA-231^MYOF-KD^ cells migrated to close the gap (Figure 3A and B and movies in Videos S1 and S2). Specifically, MDA-231^LTVC^ and MDA-231^WT^ cells exhibit significant cell-cell detachment and random, non-directional migration while closing the gap. This random cell motility pattern was also obvious from the tracks of individual cells shown in Figure 3A. Conversely, MDA-231^MYOF-KD^ cells remained as a cohesive monolayer while closing the gap and migrated in a directional, sheet-like fashion. The highly directional nature of MDA-231^MYOF-KD^ cell migration is evident from cell tracks presented in Figure 3B. Therefore, MDA-231^LTVC^ and MDA-231^MYOF-KD^ cells use two very different modes of migration to close the gap. In Figure 3, we present quantitative analysis (Figure 3C, D, E, and F) of these cell migration patterns to characterize the migratory properties of MDA-231^LTVC^ and MDA-231^MYOF-KD^ cells.

Figure 3A and 3B shows representative cell migration paths for both the MDA-231^LTVC^ and MDA-231^MYOF-KD^ cells. Note that all cells were initially located at the origin and the compact nature of the tracks in Figure 3B indicate that MDA-231^MYOF-KD^ cells move in a coordinated fashion while the disperse nature of tracks in Figure 3A indicate that MDA-231^LTVC^ cells move in an uncoordinated, random fashion. Analysis of data similar to that shown in Figures 3A and 3B indicates that MDA-231^MYOF-KD^ cells migrate with an average cell velocity that was less than one-half of that observed in MDA-231^LTVC^ control cells (Figure 3C, p<0.001). Indeed, as shown in Figure 3D, analysis of directionality via Eqn (2) indicates that MDA-231^MYOF-KD^ cells migrated two-fold more directionally compared with MDA-231^LTVC^ cells (p<0.001). Interestingly, the total distanced traveled (i.e. the accumulated distance) by MDA-231^MYOF-KD^ cells is significantly lower than the total distance traveled by MDA-231^LTVC^ cells (Figure 3E, p<0.001). However, the Euclidean distance traveled (i.e., the distance between the start and end of a cell track) for both MDA-231^LTVC^ and MDA-231^MYOF-KD^ cells were very similar (Figure 3F).

This suggests that although MDA-231^MYOF-KD^ cells migrate with a slower average velocity, their migration paths are more directed while MDA-231^LTVC^ has a higher migration velocity but a less directed migration pathway. Therefore, it is clear that silencing MYOF results in a dramatic change in cell motility where MDA-231^LTVC^ cells rapidly detach from the initial monolayer and exhibit a faster but non-directional mesenchymal motility pattern. Conversely, MDA-231^MYOF-KD^ cells migrate in a collective fashion, and, although their overall migration velocity is slower, this migration pattern is highly directional and therefore exhibits a more epithelial motility pattern.

### Myoferlin-depleted Cells Exhibit Enhanced Cell-substrate Adhesion

**Figure 4:**
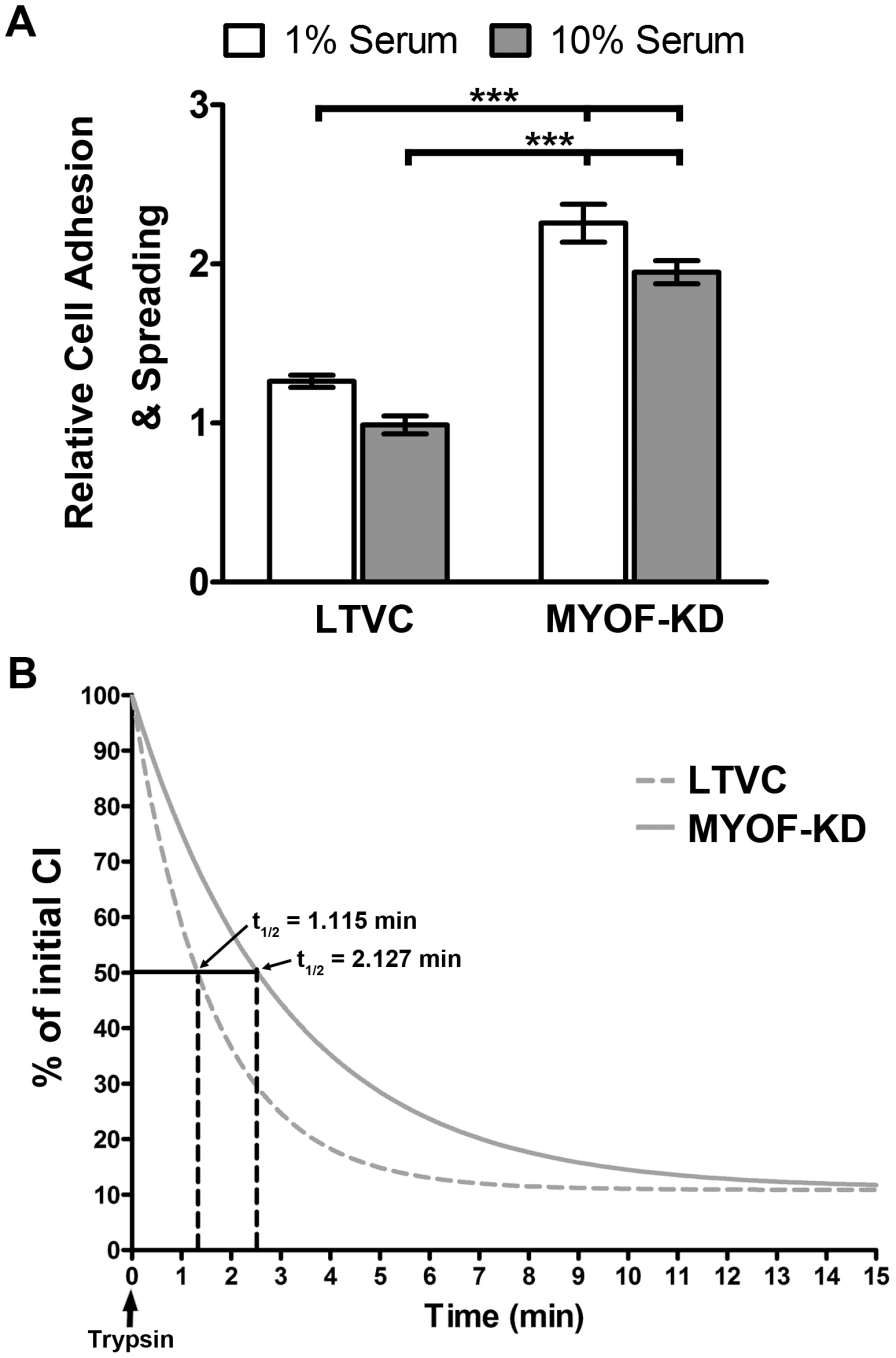
MYOF-depleted MDA-MB-231 cells exhibit enhanced cell-substrate adhesion. (A) Relative cell adhesion and spreading (RAS) was calculated from CI measurements at 3 h after plating as described in Materials and Methods in the presence of 10% (grey columns) and 1% (white columns) serum. Normalization was done with wild type cells (data not shown). The 231^MYOF-KD^ cells exhibited a two-fold greater cell adhesion and spreading than the 231^LTVC^ cells. Statistical significance (one-way ANOVA with Tukey multiple comparisons test) was between the starred bar (mean ± SEM) and the other two cell types shown, where *p<0.05, ***p<0.001. (B) Non-linear regression curve of cell detachment following addition of trypsin as calculated by normalization to the cell index immediately prior to trypsin input. The latency to release (t_1/2_) of 231^MYOF-KD^ cells was approximately two-fold greater than that of MDA-231^LTVC^ cells. MDA-231^WT^ cells gave a CI curve nearly identical to that of the lentiviral-control cells (data not shown).

To investigate how MYOF-depletion influences the adhesion properties of breast cancer cells, we measured cell spreading and substrate attachment in 2D cultures using a novel cell adhesion bioassay developed in our laboratory. Briefly, MDA-231^WT^, MDA-231^MYOF-KD^ and MDA-231^LTVC^ breast tumor cells were seeded into 16-well E-plates engineered with gold electrodes on the bottom of wells for use with the xCELLigence platform to measure electrical resistance (transcellular impedance). The cell index (CI) was collected every five minutes for four hours by the equipment according to Eqn (3), and then the relative adhesion and spreading (RAS) was calculated according to Eqn (4) with the CI at the three hour time point. Results indicate that the loss of MYOF via shRNA^MYOF^ lentiviral knockdown caused a statistically significant increase in adhesion strength as revealed by an increase in RAS of MYOF depleted cells (Figure 4A). The experiment was performed with both normal levels of serum (10%) and low serum (1%) in preparation for the trypsin-release assay, which required a low serum environment to minimize inactivation of trypsin. Relative adhesion and spreading of MDA-231^MYOF-KD^ remained much higher than MDA-231^LTVC^ cells in low serum cultures.

We also used a trypsin-release assay to measure the adhesion strength of MDA-231^WT^, MDA-231^LTVC^ and MDA-231^MYOF-KD^ cells. This method also utilized the xCELLigence RTCA system to monitor CI as a function of time. However, after seeding cells into microwells in low serum (1% FBS), cells were allowed to grow for 2 days to establish firm adhesions, and the experiments were then initiated by addition of trypsin/EDTA directly into the wells at a final concentration of 0.02% trypsin. The RTCA device then took CI measurements for two hours at 10-sec intervals to measure the drop in CI as cells released as a result of the trypsin treatment. CI measurements for each cell type were normalized to their respective CI reading immediately prior to the trypsin treatment and plotted as percentage of that initial CI over time. As shown in Figure 4B, detachment of cells under these conditions resulted in an exponential decay in CI observable in the first 15 min after the addition of trypsin. MDA-231^LTVC^ cells exhibited a faster decay in CI as compared to MDA-231^MYOF-KD^ cells indicating that silencing MYOF results in a stronger cell-substrate adhesion. The latency to release (t_1/2_) was calculated as the time it required for the CI to reach 50% of its initial value. The average t_1/2_ for MDA-231^LTVC^ control cells was 1.115 min (*95*% *confidence interval:* 1.078–1.154 min). By contrast MYOF-depleted MDA-231 cells exhibited a nearly two-fold increase in adhesion strength with a t_1/2_ = 2.127 (*95*% *confidence interval:* 2.088–2.167) (Figure 4B).

### Myoferlin-depleted MDA-MB-231 Cells Exhibit an Altered Cytoskeletal Structure, Reduced Cell Stiffness and Reduced Contractility

**Figure 5:**
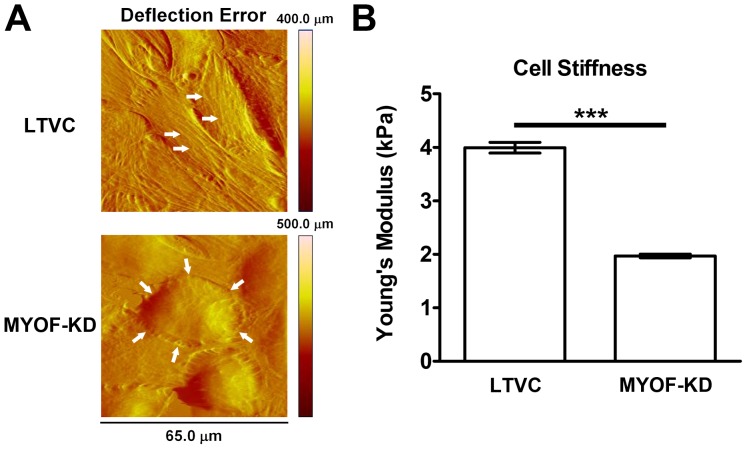
MYOF-depleted MDA-MB-231 cells result in a softer more deformable cell. The 231^LTVC^ cells (A) have an elongated morphology accompanied by significant actin stress fiber formation when compared to the more rounded 231^MYOF-KD^ cells. Cell stiffness measurements (mean ± SEM) by AFM demonstrate (B) reduced cell stiffness in myoferlin deficient cells. Statistical significance (two-tailed Mann Whitney U-test) was between the two cell types shown, where ***p<0.001.

Data in Figures 2 and 3 clearly indicate that MYOF depletion alters the migratory phenotype of MDA-MB-231 breast cancer cells. Since cytoskeletal mechanics is known to play a critical role in determining cancer cell migratory phenotype [4], [6], [7], [32], [33], we used AFM and TFM to investigate how MYOF depletion alters cytoskeletal structure, intrinsic stiffness and contractile force generation. First, AFM imaging was used to investigate how MYOF depletion affected the integrity of the cytoskeletal network. These images clearly indicated a significant difference in the actin cytoskeleton architecture of MDA-231^LTVC^ and MDA-231^MYOF-KD^ cells (Figure 5A). Specifically, the MDA-231^LTVC^ cells exhibited an elongated morphology with significant actin stress fiber formation, while MDA-231^MYOF-KD^ cells were more rounded with significantly fewer actin stress fibers and an expanded cortical actin cytoskeleton. We also used indentation AFM to measure the Young’s Modulus of MDA-231^LTVC^ and MDA-231^MYOF-KD^ cells as a measure of cell stiffness. As shown in Figure 5B, MDA-231^MYOF-KD^ cells exhibited a two-fold decrease in Young’s modulus compared to MDA-231^LTVC^ cells (p<0.001). Therefore, MYOF depletion results in a significantly softer more deformable cell.

**Figure 6:**
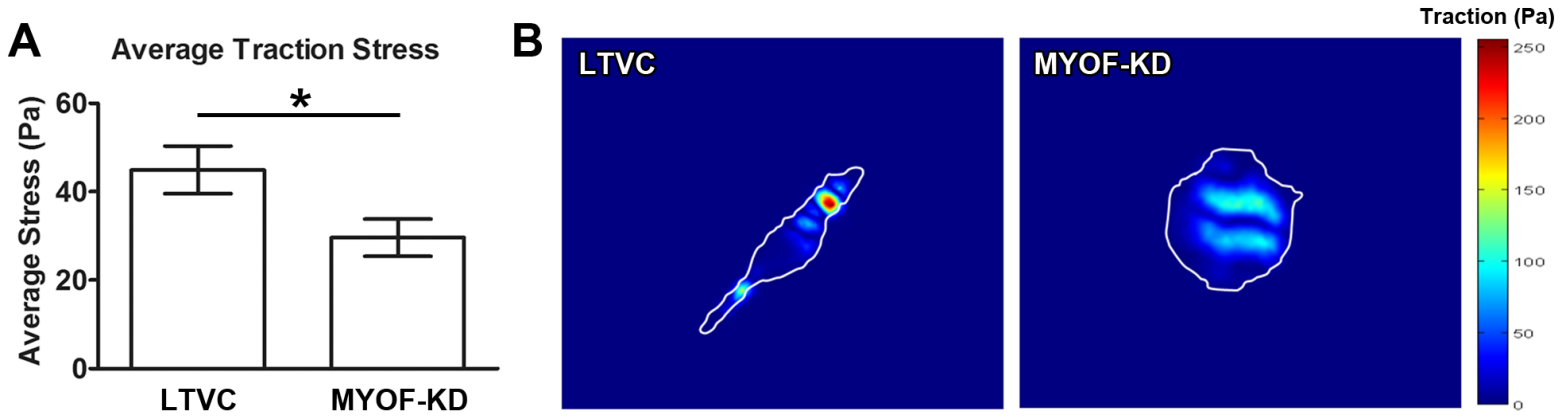
Average traction stress and contractility decreases when MYOF is depleted from MDA-MB-231 cells. Myoferlin-depleted cells exert (A) less average traction stress (mean ± SEM) on the substrate than the MDA-231^LTVC^ cells. (B) The MDA-231^MYOF-KD^ cells were more rounded cells with a more consistent traction stress over the cell area, whereas the MDA-231^LTVC^ cells were more fibroblastic morphologically with a larger range of traction stress. Statistical significance (two-tailed Student’s t-test) was between the two cell types shown, where *p<0.05.

Finally, we used TFM to investigate how depletion of MYOF alters the traction stress generated by MDA-MB-231 tumor cells. For these studies, cells were plated at sparse density to reduce error when analyzing the fluorescent microbead displacements used to calculate the traction stress in the substrate. TFM measures the traction stresses exerted by the cell on the underlying substrate as shown in Figure 6. These raw data were quantified by calculating the average traction stress exerted by individual cells according to Eqn (6). The average traction stress exerted by MDA-231^MYOF-KD^ cells was significantly lower (Figure 6A, p<0.05) than the traction stress exerted by MDA-231^LTVC^ cells. The more rounded MDA-231^MYOF-KD^ cells exert less traction on the substrate than MDA-231^LTVC^. We also noted that the MDA-231^LTVC^ cells included a larger range of traction stresses, whereas the MDA-231^MYOF-KD^ cells maintained a more consistent traction stress over the cell area (Figure 6B). Since contractile force generation by internal actin-myosin interactions is related to the amount of traction stress exerted by the cell, our data indicate that depletion of MYOF resulted in a significant reduction in the cell’s contractility or contractile force generation.

### MDA-MB-231^MYOF-KD^ Cells Form Smaller, Less Invasive Tumors in Mouse Xenografts

**Figure 7:**
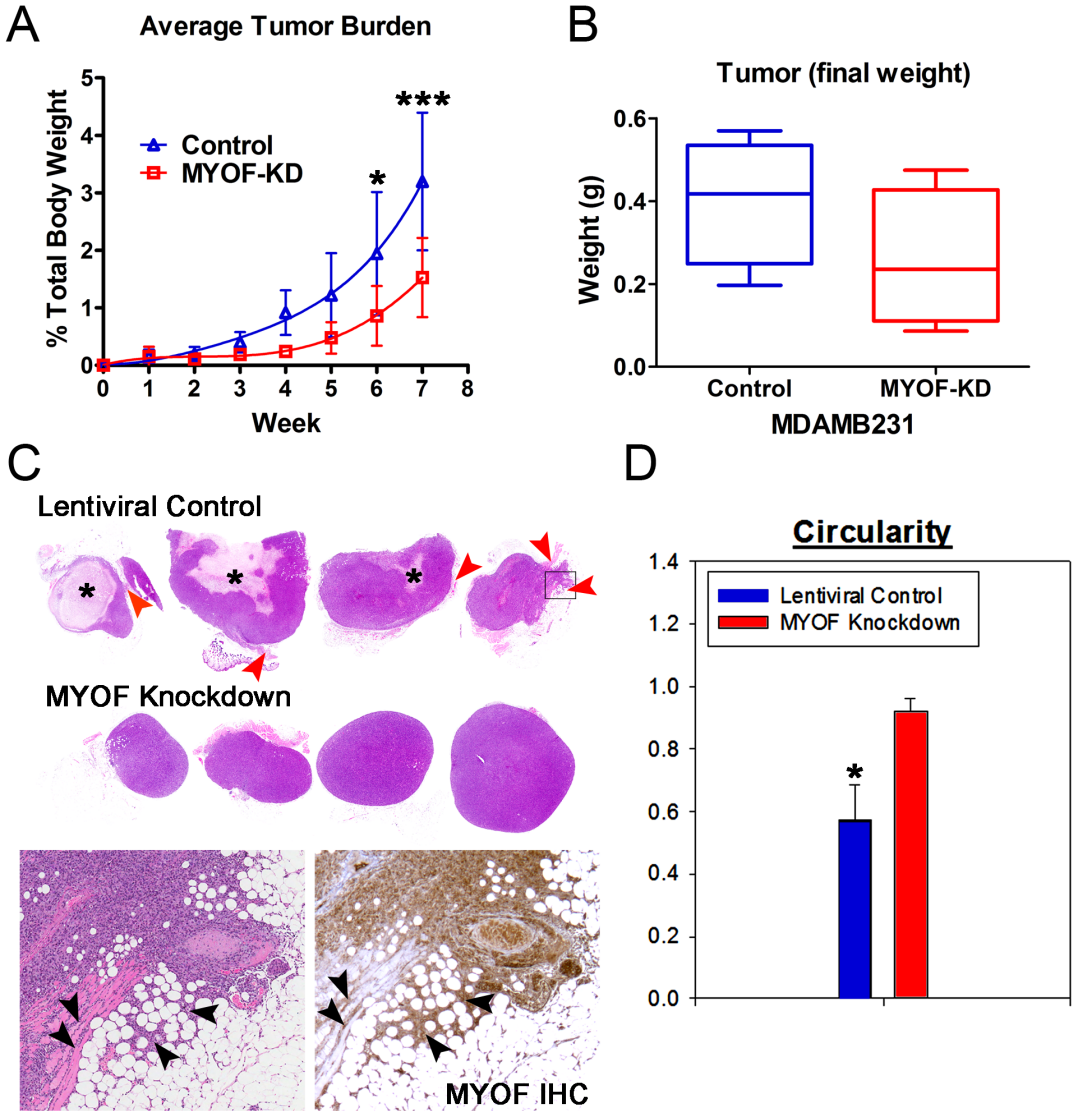
Murine xenograft model of MDA-231^LTVC^ and MDA-231^MYOFKD^ cells studying tumor growth and invasion. The excised tumors were used to study (A) tumor burden, (B) tumor mass, (C) H&E staining of tumor invasion (red/black arrows) and necrosis (black asterisks), and (D) tumor circularity. Tumor burden and circularity were statistically significant (two-way ANOVA with Bonferonni *post hoc* testing, *p<0.05, ***p<0.001) between MDA-231^LTVC^ and MDA-231^MYOFKD^ cells, with MYOF deficient tumors smaller and more circular. The bottom inserts in (C) are higher magnification of the rightmost control group tumor with H&E staining (left) and MYOF by immunohistochemistry (IHC, right) showing local stroma and muscle invasion.

The *in vitro* data presented above indicate that depletion of MYOF in the highly invasive MDA-MB-231 breast cancer cell line results in an epithelial phenotype that undergoes less detachment and a more collective/directional cell migration pattern, and that this change in migration phenotype is governed by changes in the cell’s biomechanical and biophysical properties. To validate these *in vitro* results, we have used a classic xenotransplantation model to determine whether loss of MYOF altered cell migration and invasion properties *in vivo*. For these studies, we transplanted MDA-231^MYOF-KD^ cells or their MDA-231^LTVC^ counterparts into immunocompromised mice. Four out of five mice in both groups had detectable tumors at the time necropsy (7 weeks after initial injections) and the average tumor burden was significantly lower (Figure 7A, p<0.001) in mice that received the MDA-231^MYOF-KD^ cells compared to those that recieved MDA-231^LTVC^ cells. In addition, the final tumor weight was less in the mice that received MDA-231^MYOF-KD^ cells compared with those that received MDA-231^LTVC^ cells (Figure 7B). Histological examination showed that control mice injected subcutaneously with MDA-231^LTVC^ cells developed tumors with heterogeneous morphologies, including necrotic areas (asterisks in Figure 7C) and irregular borders, suggesting local invasion. Furthermore, these tumors contained MYOF immunoreactivity within the surrounding connective tissue, further indicating local invasive activity of implanted human tumor cells (Figure 7C). In contrast, when MYOF was depleted with RNAi, implanted breast cancer cells produced smaller, smooth-bordered tumors that did not appear to invade into the local adventia (Figure 7C). No MYOF-immunoreactivity was detected in mice that received MDA-231^MYOF-KD^ cells. This change in tumor morphology was quantified by calculating the circularity according to Eqn (7) and results indicated than tumors in the mice that received MDA-231^MYOF-KD^ cells were significantly more circular and regular that the tumors in mice that received MDA-231^LTVC^ cells (Figure 7D, p<0.05).

## Discussion

Previous work by our group demonstrated a pleiotropic role for MYOF in regulating human breast cancer cell shape, motility and invasion *in vitro* [21], [34]. During early studies, we discovered a positive correlation between MYOF expression and invasive phenotype in several well-characterized breast cancer cell lines [34]. MDA-MB-231 cells in which MYOF was knocked down using a lentivirus shRNA targeting vector showed diminished expression of several matrix metalloproteinases (MMPs) and failed to efficiently invade through Matrigel or collagen type I [21]. Mathematical models were consistent with these experimental findings and further showed decreased activation of several receptor tyrosine kinases (RTKs) [34]. This latter observation is similar to a report by Bernatchez et al. who demonstrated that loss of MYOF resulted in impaired vascular endothelial growth factor receptor (VEGFR) and angiopoietin receptor (Tie-2) signaling in endothelial cells [25], [26], [35]. Separately, McNally’s group reported that insulin-like growth factor-I receptor (IGF-IR) signaling was disrupted in MYOF-null mice leading to impaired skeletal myogenesis [36]. While it is too early to define a unifying mechanism to explain how MYOF controls RTK activity and MMP expression, a role for receptor stabilization has been suggested by Bernatchez et al. [26]. The results from these combined investigations point to a critical role for MYOF in membrane receptor signaling and MMP production and secretion, potentially contributing to tumor cell chemotaxis and invasion [12].

Although biochemical approaches are important for defining the molecular details of MYOF function in the context of cancer, increasing emphasis is being directed toward a more holistic set of strategies that include an appreciation of the mechanical principles of tumor cell biology [37]–[40]. This comprehensive approach to understanding cancer cell motility is exemplified by the recently established Physical Sciences-Oncology Centers Network (PS-OCN). As such, in the current series of studies, we focused on the mechanobiology of breast cancer cell motility and have shown that, in addition to its role in tumor cell invasion, MYOF governs undirected, single cell migration reminiscent of mesenchymal motility and that depletion of MYOF results in collective cell migration reminiscent of epithelial sheet–like migration [5], [20], [41]–[44]. Furthermore, we demonstrated that this switch from a random motility phenotype to a collective migration pattern is accompanied by distinct changes in tumor cell biomechanical and biophysical properties. Finally, we demonstrated that MYOF depletion also significantly affects tumor growth and progression *in vivo*.

The initial steps of metastasis typically involve the detachment of cells from the primary tumor and the enhanced single cell migration/invasion of cells through the tumor microenvironment. In this study, we used an Ibidi cell culture insert and live-cell tracking routines to evaluate the migratory properties of MDA-MB-231 breast cancer cells with shRNA-mediated knockdown of MYOF and cells treated with a lentiviral non-human, non-targeting control vector. The Ibidi insert method coupled with live-cell microscopy for studying cell migration has significant advantages over standard scratch wound healing assays, because it allows for the formation of a well-defined “wound edge” without physically damaging the cells and greatly facilitates the evaluation of collective versus single cell motility patterns. In addition, this system can mimic the cell detachment processes that occur during the initial steps of metastasis, albeit in two dimensions. In this study, we showed that after removal of the Ibidi insert, the lentiviral control MDA-MB-231 breast cancer cells rapidly detach from the monolayer and acquire a single cell, mesenchymal-like motility pattern. In contrast, shRNA-mediated knockdown of MYOF resulted in a much slower, highly directed migration. Interestingly, even though the MDA-231^MYOF-KD^ cells migrated at one-half the velocity of MDA-231^LTVC^ cells, both cell types closed the “wound” gap in the same time interval. Therefore, the use of standard percent wound closure measurements would not have been able to detect the significant differences in cell migration properties that were readily detected with our technique.

Another interesting feature we observed in MYOF-depleted MDA-MB-231 cells was their propensity for collective migration. This was not completely surprising, inasmuch as MDA-231^MYOF-KD^ cells re-expressed E-cadherin and became highly epithelioid in morphology [21]. However, collective migratory behaviors have been reported to occur in a ‘physical and mechanical nature’ as suggested by Rorth [45] even with the lack of fully demonstrable cell-cell contacts. Moreover, Vedel et al. [46] demonstrated that, even when not directly cohesive, collective migration can occur through local influences on neighboring cells. Importantly, *in vivo* experiments confirmed the migratory patterns observed *in vitro*. Specifically, tumors from mice injected with MDA-231^LTVC^ cells exhibited an irregular morphology and decreased circularity indicative of cell detachment and invasion into the surrounding extra-cellular matrix. In contrast, tumors from mice injected with MDA-231^MYOF-KD^ cells remained highly circular and had smooth margins that is consistent with collective cell migration and growth to maintain a more regular tumor morphology.

In this study, we also demonstrated that MYOF knockdown resulted in distinct changes in cellular biomechanical properties. Importantly, as described below, these changes in mechanical properties can be used to explain the biophysical mechanisms responsible for the switch from a random mesenchymal motility pattern to a collective epithelial motility pattern. First, we demonstrated that MDA-231^MYOF-KD^ cells have significantly stronger cell-substrate adhesions than MDA- 231^LTVC^ cells. Increased cell-substrate adhesion is consistent with the decreased migration velocity observed in MDA-231^MYOF-KD^ cells. In addition, although we did not directly measure cell-cell adhesion strength, as shown in our previous studies, the re-expression of E-cadherin, likely leads to enhanced cell-cell adhesion. This would, in turn, facilitate the collective cell migration pattern observed in MDA-231^MYOF-KD^ cells.

We also used AFM force-indentation techniques to demonstrate that the Young’s modulus, a measure of cell stiffness, in MDA-231^MYOF-KD^ cells is one-half the value in MDA- 231^LTVC^ cells. Given the decreased migration velocity of MDA-231^MYOF-KD^ cells, we were initially surprised by this decrease in cell stiffness, because decreased cell stiffness has been associated with increased cancer cell motility and the metastatic phenotype [47]–[50]. However, a majority of the previous studies have been performed on isolated single cells. Under these conditions, amoeboid motility would dominate and softer cells would be able to more easily squeeze through pores in the extracellular matrix. By contrast, our study considered the initial migration of cells within and away from an intact monolayer (a 2D analogue to an intact tumor). Under these conditions, traction forces and the resulting tensile stress development within the monolayer has been shown to govern collective cell migration [6]. Therefore, in addition to measuring cell stiffness, we used TFM techniques to measure the traction stresses generated by MDA-231^MYOF-KD^ and MDA- 231^LTVC^ cells. These results clearly indicated that MDA-231^MYOF-KD^ cells generated significantly less traction stress than MDA- 231^LTVC^ cells. We conclude that the decreased migratory capacity after MYOF depletion is primarily governed by a reduction in the contractile forces and traction stresses generated by the MDA-231^MYOF-KD^ cells. Previous investigators have also demonstrated that less invasive/metastatic cancer cells exhibit a reduced traction stress [33], and future studies that investigate the intracellular signaling cascades that govern this reduction in traction stress are warranted.

To address whether MYOF loss could result in altered *in vivo* tumor formation, we transplanted MDA-231^MYOKD^ cells into nude mice to examine the role that MYOF may play in tumor growth and early invasion. MYOF-deficient cells when implanted into immunodeficient mice failed to form locally invasive tumors. Rather, they were highly circularized and did not invade into the local adventitia. In contrast, tumors derived from MDA-231^LTVC^ cells formed larger, irregular masses with a very heterogeneous cancer cell population. Immunohistochemical staining with anti-human MYOF demonstrated that MYOF-positive tumor cells in control mice were found in the surrounding adipose and connective tissues to create tumors with jagged edges. We did not detect MYOF-immunoreactivity in the MYOF knockdown cells supporting our earlier data showing >95% depletion (data not shown). Therefore, the effect of MYOF depletion on cell migration observed *in vitro*, including reduced cell detachment and a switch to collective epithelial-like cell migration, correlate with our *in vivo* observations of decreased invasive processes and a more regular/circular tumor morphology.

The Ibidi live-cell imaging system was used to study the effect of MYOF depletion on cell migration. However, this 2D monolayer system does not account for important 3D features of cancer cell migration including degradation of the tumor stroma and extracellular matrix via MMPs and the amoeboid motility of cells through pores in the ECM. Although future studies will focus on the development of more realistic *in vitro* models of tumor cell migration in 3D, we note that, in this study, migratory behaviors observed in our simple 2D *in vitro* model were correlated with tumor morphogenesis measurements *in vivo*. We also used a sharp AFM tip to obtain measures of the overall stiffness of the cell. Although these AFM probes are well-suited for high-throughput analysis of cell mechanics, they do not provide sufficient resolution to determine if our data measured strictly membrane or cytoskeletal stiffness, or both. Future studies could either use low-throughput spherical indenters or a combination of shallow and deep indentations to more accurately measure plasma membrane and cytoskeletal tension independently. Recent work by Diz-Munoz et al. [32] has shown that cell stiffness within the plasma membrane and underlying cytoskeleton can lead to alterations in membrane tension and the formation of leading edge protrusions. Although tension from the membrane and cytoskeleton act together to limit leading edge protrusions [32], [51]–[53], our current work did not address the molecular mechanisms that underpin the mechanical alternations we observed when MYOF was disrupted in cancer cells. Therefore, future studies should investigate the role of MYOF in controlling intracellular signaling cascades that govern cell motility [54] and local changes in plasma membrane lipid composition [53].

The *in vitro* biomechanical and *in vivo* tumor formation studies outlined in the current work shed new light on a novel role for MYOF in cancer cell motility and invasion. A recent report by Leung et al. [55] showed that breast and lung cancers express high levels of MYOF and that knockdown with siRNA leads to decreased tumor cell proliferation. In addition, this same group also revealed that loss of MYOF decreased tumor burden in a mouse xenograft model. These data are in agreement with our current findings.

Increasing interest has shed light on the potential to target one or more elements of the invasive and metastatic cascade [43]. That MYOF plays a pleiotropic role in breast cancer cell migration and invasion, and that these data have now been translated to an animal model of human cancer offers the potential to use MYOF as a biomarker of disease progression, invasion and impending metastasis. Furthermore, it may be possible to target MYOF expression or develop small molecule inhibitors of MYOF function as a novel chemotherapeutic agent used as primary or adjuvant therapy.

## Supporting Information

Figure S1 **Genetic map of the lentivirus vector encoding human MYOF short-hairpin RNA.** (A) shRNA^MYOF-KD^ or (B) shRNA^LTVC^ non-targeting (control) vectors were selected in puromycin, and positive cultures were evaluated for MYOF mRNA and protein expression by qRT-PCR and immunoblotting, respectively. MYOF expression at the mRNA and protein levels were diminished by >95%, and the phenotype has been stable for >150 serial passages. Cells were archived in liquid N_2_ until used for experiments.
(TIFF)

Video S1 **Movie of the MDA-231^LTVC^ cells (arrows) migrating for 24 h during wound closure.** Images were collected every 10 min for 24 h following removal of the Ibidi® chamber inserts. Note that MDA-231^LTVC^ cells exhibit a random pattern of individual cell migration reminiscent of a mesenchymal phenotype. Scale bar = 20 µm.
(AVI)

Video S2 **Movie of the MDA-231^MYOF-KD^ cells (arrows) migrating for 24 h during wound closure.** Images were collected every 10 min for 24 h following removal of the Ibidi® chamber inserts. Note that the MDA-231^MYOF-KD^ cells move in a collective migration pattern reminiscent of epithelial cells. Scale bar = 20 µm.
(AVI)
