# Supplementary figures and images for: Loss of Myoferlin Redirects Breast Cancer Cell Motility towards Collective Migration
Source: PLoS One. 2014 Feb 26;9(2):e86110. doi: 10.1371/journal.pone.0086110 (PMC3935829; doi:10.1371/journal.pone.0086110)

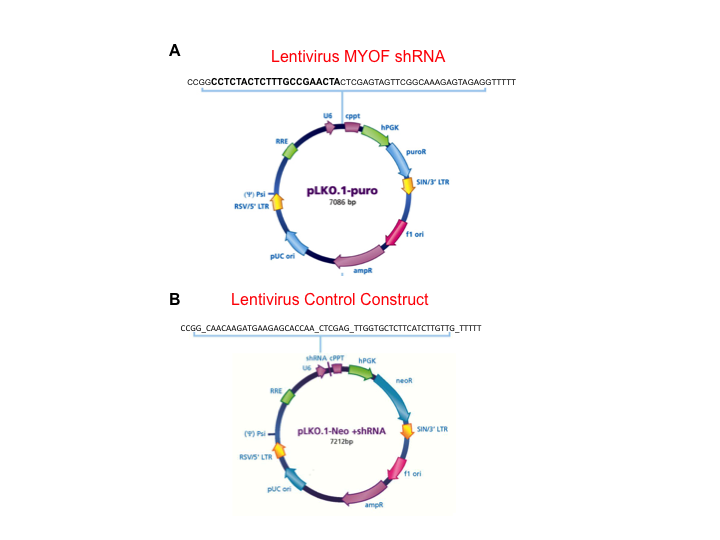

Supplement: Figure S1 — Genetic map of the lentivirus vector encoding human MYOF short-hairpin RNA. (A) shRNAMYOF-KD or (B) shRNALTVC non-targeting (control) vectors were selected in puromycin, and positive cultures were evaluated for MYOF mRNA and protein expression by qRT-PCR and immunoblotting, respectively. MYOF expression at the mRNA and protein levels were diminished by >95%, and the phenotype has been stable for >150 serial passages. Cells were archived in liquid N2 until used for experiments. (TIFF) [file pone.0086110.s001.tiff]
